# Using Biomarkers to Inform Cumulative Risk Assessment

**DOI:** 10.1289/ehp.9334

**Published:** 2007-01-24

**Authors:** P. Barry Ryan, Thomas A. Burke, Elaine A. Cohen Hubal, Jerome J. Cura, Thomas E. McKone

**Affiliations:** 1 Department of Environmental and Occupational Health, Rollins School of Public Health, Emory University, Atlanta, Georgia, USA; 2 Department of Chemistry, Emory University, Atlanta, Georgia, USA; 3 Department of Health Policy and Management, Johns Hopkins University, Baltimore, Maryland, USA; 4 U.S. Environmental Protection Agency, Research Triangle Park, North Carolina, USA; 5 Menzie-Cura & Associates, Winchester, Massachusetts, USA; 6 Department of Environmental Health, University of California, Berkeley, Berkeley, California, USA

**Keywords:** biomarkers of effect, biomarkers of exposure, biomarkers of susceptibility, cumulative exposure, multiple stressors, risk assessment

## Abstract

**Background:**

Biomarkers are considered the method of choice for determining exposure to environmental contaminants and relating such exposures to health outcomes. However, the association between many biomarkers and outcome is not direct because of variability in sensitivity and susceptibility in the individual.

**Objectives:**

We explore the relationship between environmental exposures and health outcomes as mitigated by differential susceptibility in individuals or populations and address the question “Can biomarkers enable us to understand and quantify better the population burden of disease and health effects attributable to environmental exposures?”

**Methods:**

We use a case–study approach to develop the thesis that biomarkers offer a pathway to disaggregation of health effects into specific, if multiple, risk factors. We offer the point of view that a series or array of biomarkers, including biomarkers of exposure, biomarkers of susceptibility, and biomarkers of effect, used in concert offer the best means by which to effect this disaggregation. We commence our discussion by developing the characteristics of an ideal biomarker, then give some examples of commonly used biomarkers to show the strengths and weaknesses of current usage. We follow this by more detailed case-study assessment outlining the state-of-the-science in specific cases. We complete our work with recommendations regarding the future use of biomarkers and areas for continued development.

**Conclusions:**

The case studies provide examples of when and how biomarkers can be used to infer the source and magnitude of exposure among a set of competing sources and pathways. The answer to this question is chemical specific and relates to how well the biomarker matches the characteristics of an “ideal” biomarker–in particular ease of collection and persistence. The use of biomarkers in combination provides a better opportunity to disaggregate both source and pathway contributions.

The U.S. Environmental Protection Agency (EPA) defines cumulative risk as the combined risks from aggregate exposure to multiple stressors (U.S. EPA 2003), and cumulative risk assessment as an analysis, characterization, and possible quantification of these risks. In this and companion papers (Callahan and Sexton 2007), we explore approaches for assessing the effects of stressors from multiple sources (Menzie et al. 2007), the effects of differential vulnerability in populations and individuals (deFur et al. 2007), and the effects associated with differential exposure (Sexton and Hattis 2007).

This article provides an evaluation of whether and how biomonitoring data can inform cumulative risk assessment. We examined the potential for human and ecosystem biomarkers to help us understand cumulative health risks from the interactions between environmental exposures and host susceptibility factors. We used case studies to address the question “Can biomarkers enable us to understand and quantify better the population burden of disease and health effects attributable to environmental exposures? Further, we present examples from the current literature on the availability and uses of biomarkers to explore two other questions:

- Under what circumstances can biomarkers be used to disaggregate disease burden into specific risk factors? For example, when and how can biomarkers be linked to specific diseases, and can a specific biomarker or set of biomarkers be useful for mapping disease to exposure?
- When and how can biomarkers be used to infer the source and magnitude of exposure among a set of competing sources and pathways?

Specific health outcomes potentially may come from numerous differing exposures. The problem of trying to infer which exposures lead to these outcomes is not simple. The first of these two questions focuses on assessing multiple risk factors that may lead to health outcomes, whereas the latter question centers on disaggregating cumulative exposures into their component parts. In this article, we use the term “disaggregation” to address both of these issues, namely, risk factors leading to a health outcome and exposures leading to a health outcome. These are difficult problems that are only now receiving attention in the scientific community.

Biomarkers may offer improved understanding of the pathway between the causative agents, as indicated by exposure measures, and the health outcome. There are many challenges and limitations. What are the public health implications of widespread low-level population exposures that can now be inferred from biological or ecologic measurements? Disaggregating the health effects and understanding the health risk from these exposures will require new perspectives on the environmental health paradigm. Ideally, biomonitoring will become a foundation of an environmental public health tracking system that includes identification of environmental sources, exposures, and related population health outcomes (Barr et al. 2005a). Combining biomonitoring results with population health surveillance offers opportunities for understanding the relationship between cumulative exposures and population, community, and individual risk.

## Biomarkers As a Possible Basis for Environmental Regulation

Medical surveillance studies of disease burdens provide insight on cumulative impacts from multiple risk factors but do not provide a framework to attribute risk factors to disease. This attribution is necessary for regulation. The problem is finding a measurable basis for regulation of disease-causing agents that provides a quantitative or qualitative link or links between disease and its causative agent(s) in the environment. Biomarkers in human and ecologic populations may provide this basis. Biological exposure indices (BEIs) and biological tolerance values (BATs) have been used in the industrial hygiene community for decades in an effort to protect workers (Plog et al. 1996); however, only recently have analytical measurements become sensitive enough to allow quantification of biological measures in the nonoccupational setting. Thus it has now become feasible to develop in a framework upon which to build a better understanding of the relationship between such measures and exposures in the community setting.

We define biomarkers as measures reflecting an interaction between a biological system and a chemical, biological, or physical environmental agent. The focus is on chemical agents, although the methods developed are applicable in other areas. The literature usually considers three classes of biomarkers [National Research Council (NRC) 1987; World Health Organization (WHO) 1993]: biomarkers of exposure, biomarkers of effect, and biomarkers of susceptibility. The first two link exposures with health outcomes and can provide the basis for linking biological effect and exposure to environmental contamination. The third refers to a modifier that influences the magnitude of or induces the effect given a fixed magnitude of the driver. All three types may be used to identify vulnerable individuals or populations. Biomarkers of exposure may include measurements of parent compounds, metabolites, or DNA or protein adducts of parent compounds and/or metabolites that indicate that a direct exposure to the compound of interest has occurred. Biomarkers of effect may include measurements of changes in the biological system, e.g., acetylcholinesterase inhibition associated with organophosphate pesticide exposures or changes in cytochrome P450 enzyme activity. Biomarkers of susceptibility may include, for example, polymorphisms in specific genes associated with metabolism of toxic material in the body.

### Characteristics of an ideal biomarker

We used the characteristics of an ideal biomarker to evaluate the current state of research, guide the use of specific biomarkers, and to suggest future research. The literature on the criteria for defining, developing, and validating an ideal biomarker (Groopman and Kensler 2005; Metcalf and Orloff 2004; Schulte and Mazzuckelli 1991; WHO 1993) suggests seven criteria for evaluating a biomarker’s usefulness in cumulative risk assessment. Ideal biomarkers would:

- be persistent—have a long half-life;
- be easily collected—collected using non-invasive procedures that present only minor procedural difficulties in collection, transport, storage, and analysis;
- be linked to disease—display exposure, indicate effect, and establish a link between them;
- have a large sample—to examine the distribution of the biomarker in the population and to establish links between the bio-marker and effect, it is important that the biomarker be found in a substantial fraction of the population;
- have broad spatial distribution and temporal occurrence—a complete spatial and temporal understanding of the exposure/health outcome distribution;
- have sensitivity—sufficiently sensitive to give information on differences in populations from different regions and over time scales of interest, for example, seasonal or long-term, secular trends; and
- favor measurement of parent compounds over metabolites—the measurement of parent compound gives a direct and unambiguous measure of exposure to the contaminant of interest.

Table 1 presents an outline of considerations for biomarkers, including a hypothetical biomarker that illustrates the criteria. Table 1 shows that even commonly used biomarkers are far from ideal but that combinations of biomarkers for the same compound may give complementary information.

## An Array of Biomarkers in Cumulative Risk Assessment

Although a biomarker of exposure may be appropriate in assessing exposures over one time scale, health outcomes, and thus risk, may be associated with time scales markedly different from those being assessed. Further, exposure to a specific compound—a biomarker of exposure—may be insufficient to assess effects; a biomarker of effect may be needed. Finally, if one is concerned about large populations and the health impact they are likely to experience, then one must be concerned about differential susceptibility in the population; a biomarker of susceptibility is needed. Thus the “ideal” biomarker may not exist.

### Application of array concept—organophosphate pesticides

**Figure 1:**
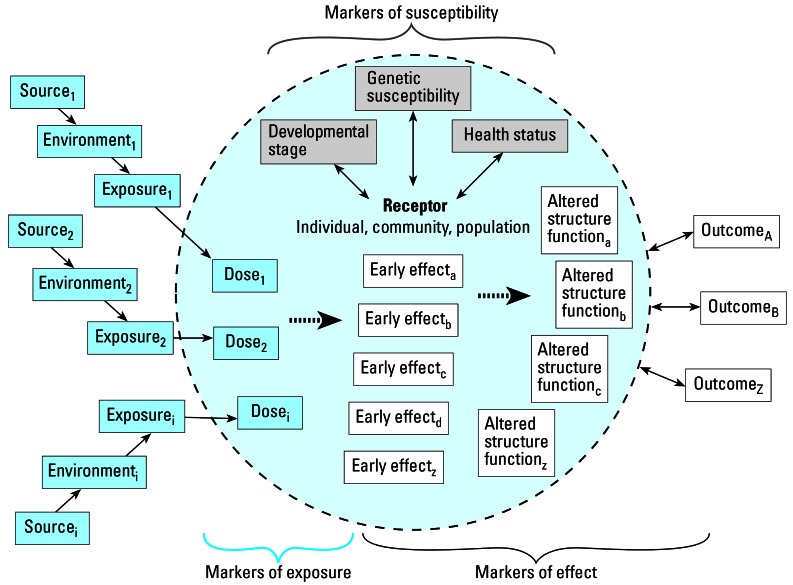
Framework for biomonitoring.

It may be possible to approach the ideal biomarker functionally by applying an array of biomarkers, each of which provides some of the ideal characteristics. For example, organophosphate pesticides (OPs) have widely varying chemical structures but share a common toxic mechanism of action: acetylcholinesterase inhibition. Although exposure to OPs has been linked to neurologic effects, this primary mechanism of action is actually a short-term, or early, effect (Figure 1).

Biomarkers of exposure to these compounds exist. Dialkyl phosphates (DAPs) (Barr et al. 2004) and OP-specific (or near-specific) biomarkers such as 3,5,6-trichloro-2-pyridinol (TCPy) (Barr et al. 2005b; MacIntosh et al. 1999) offer two approaches to assessing OP pesticide exposure. The nonspecific biomarker for OPs, DAPs, may offer insight into the general exposure to these pesticides and a pathway to cumulative risk assessment, whereas the OP-specific biomarker gives insight into exposure to specific members of this class, for example, chlorpyrifos (and chlorpyrifosmethyl) and some insight into metabolic processes. Measurement of the compound-specific bio-marker, for example, TCPy, can give information only on exposure to the compounds from which it is derived. Further, interpretation is complicated because of levels of TCPy in exposure media (Lu et al. 2006; Morgan et al. 2005; Wilson et al. 2004). Degradation of the parent compound in environmental media, including food, and subsequent intake of the degradation product may lead to overestimation of the intake of the parent compound, reducing the effectiveness of such a biomarker in inferring exposure. Although the DAPs measure gives information on all compounds of this general class, each member of the OP class has different inhibition characteristics. The measure of the nonspecific metabolites does not give information on the specific compound from which they came, reducing, although not eliminating their use.

Directly measuring acetylcholinesterase inhibition, a biomarker of effect, may add some insight into susceptibility, but it provides no information on the cause of inhibition. Recent work (Costa 2006; Costa et al. 2005) suggests that polymorphisms in paraoxonase may offer a direct biomarker of susceptibility with regard to OPs, but more research is needed to establish relationship norms for this marker. The discussion above suggests that the simultaneous collection of all three biomarkers is likely to lead to substantially more information than collection of the individual markers alone.

## A Framework for Applying an Array of Biomarkers and Other Metrics

The framework presented in Figure 1 provides the conceptual basis for considering an array of biomonitoring data and other health metrics in assessment of cumulative exposure and risk. The receptor may be a human being, an individual organism, a population, a community, or an ecosystem. Multiple sources result in environmental conditions ranging from temporal and spatial exposures to multiple stressors (Sexton and Hattis 2007). These multiple exposures at varying times may be required to induce any one outcome.

The framework uses the following bio-markers:

- biomarkers of susceptibility to characterize the receptor (e.g., information on genetics, developmental or life stage, health status, age, preexisting disease, nutritional status) (deFur et al. 2007);
- biomarkers of effect to characterize the potential for or existence or prevalence of adverse outcomes (e.g., may range in scale from early effects detectable at a molecular level to manifestations of the full disease state); and
- biomarkers of exposure to characterize direct impact of stressors on the receptor (Menzie et al. 2007).

Linking these internal markers of exposure to associated chemical, biological, physical, or psychosocial stressors often will require consideration of additional information on conditions of the environment and interaction of the receptor with the environment. In addition, it is important to link the timing, duration, and route of exposure as well as the kinetics of uptake and elimination for a given set of chemicals to the biomarker of exposure.

### Applying the framework

#### Multiple sources

Even for a specific contaminant, there is the potential for multiple sources and pathways of exposure. For example, in the case of OPs, a receptor can be exposed through various sources including ingestion of agricultural crops or direct contact from local application for pest control. Each of these sources results in environmental contamination, but the movement through the environment that produces the resulting exposure is substantially different. One may attempt to monitor the food supply, house dust, soil, and air concentrations (the exposure) and infer dose to the receptor through a modeling process that estimates intake to the body based on the amount of specific compound found in each of the media. However, such calculations are difficult and results may depend upon the model selected.

### Factors influencing effects

Three factors of interest influence the effects observed on the receptor: genetic susceptibility, developmental stage, and health status (Figure 1). Genetic susceptibility—for example, through differential metabolism of OPs among various individuals, communities, or populations—may result in different effects for a given exposure. Developmental stage is an important determinant of the effects of exposure. Perhaps the most visible case of such effects occurred in the so-called thalidomide babies born to mothers who took this drug in the early 1960s. Developmental stage was critical; those who were exposed to thalidomide at a particular stage of gestation suffered from the exposures, whereas others exposed later in gestation, or not at all, did not experience any such effects. For effects (Figure 1, right side), various levels of outcomes range from often subclinical “early effects” through altered structure and function, leading to a measured adverse outcome. It is important to note that a dose from a single chemical may lead to multiple early effects, altered structure and functions, and outcomes. For example, exposure to lead may lead to cognitive and neurologic effects (Stewart et al. 2006) and altered blood pressure status (Glenn et al. 2003). Thus, one progresses from a multifactorial source–exposure–dose relationship, modified by receptor status with respect to development, susceptibility, and health, to multifactorial outcomes.

#### Health status of the receptor

Health status of the receptor can also affect the outcome of a given exposure. Those with compromised immune systems due to disease status, or those with, for example, little excess pulmonary capacity, may be more adversely affected to a given exposure than those not suffering from these conditions. Similarly, an ecosystem under stress from, for example, ozone exposure, may respond differently to herbicides than a healthier ecosystem (Menzie et al. 2007; Sexton and Hattis 2007). The key question becomes “How do we think about the appropriate array of bio-markers needed to assess fully the outcomes of interest for the specific receptor?”

## Setting a Research Agenda

The review of biomarker data indicates that there probably is no single, ideal biomarker (e.g., Table 1), and the discussion of using an array of biomarkers to assess the cumulative effects of various contaminants, poses various established uncertainties (e.g., a full suite of biomarkers—exposure, susceptibility, and effect) may not exist for a specific compound. However, we believe that an integration of the characteristics of an ideal biomarker with existing knowledge of the specific difficulties surrounding a specific outcome of concern can offer research recommendations. This is a simple matrix that matches a set of evaluation criteria (the criteria that define an ideal biomarker) to the current state of knowledge (an array of existing biomarkers and other health criteria). One can use that knowledge to fill the matrix and specify the uncertainties associated within each match-up. Figure 1 presents the essentials of the matrix. At a minimum, the questions addressed in each element should include the following:

- Does the biomarker have the desired criteria?
- Are there multiple sources that affect the biomarker?
- Is the biomarker indicative of genetic status?
- Is the biomarker indicative of developmental stage?
- Is the biomarker indicative of health status?
- Are there many potential outcomes (e.g., multiple early effects, altered structure and functions, and changed health status)?

The answers to these questions, informed by the ideal criteria, will produce a coherent research agenda suitable to prioritization.

## Biomarkers and Disease Outcomes—Illustrative Case Studies

**Figure 2:**
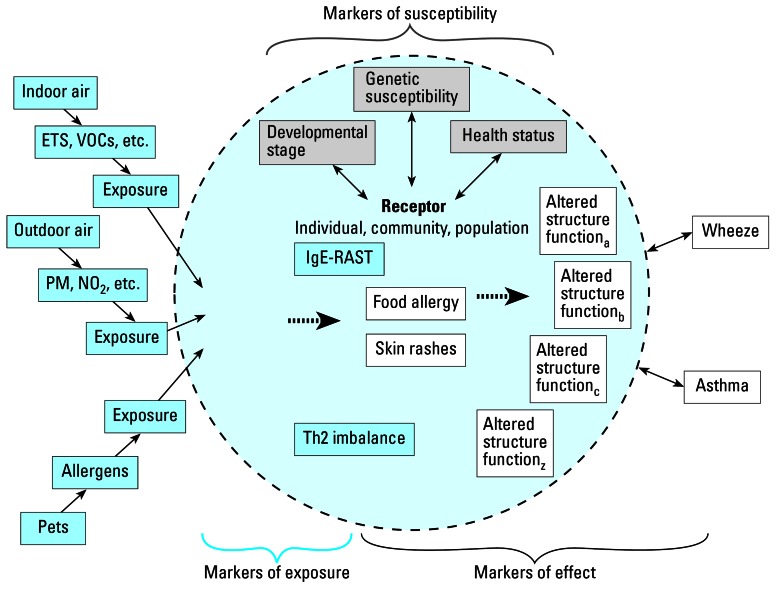
Framework applied to asthma case study. Abbreviations: ETS, environmental tobacco smoke; PM, particulate matter; NO_2_, nitrogen dioxide; RAST, radioallergoabsorbent test; Th2, T-helper 2; VOCs, volatile organic compounds.

Until now the focus has been on the exposure side, approaching biomarkers as a method of assessing exposure experienced by a receptor to some environmental contaminant. We now change the focus to outcome. We pose the question “Can the presence of certain bio-markers in the receptor aid in evaluating the source of a particular health outcome?” Two cases studies are presented on disease outcomes of concern: asthma and neurobehavioral effects. Both are complex diseases with many different causes and manifestations. Both have multiple biomarkers, including those of exposure, effect, and susceptibility, that can be used singularly or in combination to assess the contaminant sources affecting the disease and the disease outcome. We propose to evaluate multiple biomarkers simultaneously, the main proposal advocated in this work. For one of these cases, asthma, the matrix in Table 2 is applied to assess the status of each associated biomarker relative to the ideal. Also for this case, we present the graphical framework (Figure 2).

### Asthma and asthma etiology markers and effects

#### Background

Asthma and related allergic diseases (ADs) are manifested by numerous environmentally related sources, and numerous potential outcomes. In the United States, recent surveys find a 16% prevalence of asthma among children 14 years of age, with a 200% increase in the rate of asthma hospitalizations and a 100% increase in the rate of asthma mortality since the 1970s. The “atopic march” is the accepted natural history of ADs and refers to a sequence of early immunologic and later clinical responses that may appear in young children, persist over years, and may continue throughout one’s lifetime (Kulig et al. 1999). A series of biomarkers may afford an improved understanding of the effects of various sources on the etiology of these related diseases. Examination of the contaminant sources thought to influence the course of the atopic march and the biological markers associated with this process is instructive in light of the concepts presented in this article.

#### Sources and exposures

There is much evidence to suggest that components of the early childhood indoor and outdoor environments are contributing to the increasing prevalence of ADs (Figure 2). Various airborne sources are associated with the etiology of ADs and the specific development of asthma. These include: photochemical and particulate air pollution (Andrae et al. 1988; Diaz-Sanchez et al. 1997; Lunn et al. 1970; Peterson and Saxon 1996; Ussetti et al. 1984; Ware et al. 1986); criteria air pollutants, diesel exhaust carbon particles, and pesticides that increase allergic sensitization (Behrendt et al. 1997; Emberlin 1995; Knox et al. 1997; MacIntosh et al. 1999; Rubbin et al. 1986; Ruffin et al. 1986); and, elevated airborne levels of particulate matter (PM), particularly fine aerosol (particulate matter with an aerodynamic diameter ≤ 2.5 μm) (Dejmek et al. 1999; Neas et al. 1994; Norris et al. 1999; Romieu et al. 1996). Specific biomarkers for many of these pollutants are not yet available.

Individuals living in industrialized societies also tend to spend a larger fraction of time in indoor environments that have higher allergen and chemical burdens (Platts-Mills et al. 1996). There is some evidence that indoor exposure to volatile organic compounds can be related to asthmatic symptoms (Harving et al. 1991). Among infants exposed to environmental tobacco smoke, studies demonstrate that the risks of lower airway disease and recurrent wheezing are increased (Arshad and Hide 1992; Murray and Morrison 1990; Wahn and von Mutius 2001). Exposure to household endotoxin may also be important in the development of ADs. There is evidence that high levels of household endotoxin may mitigate the development of ADs before disease onset but may exacerbate the symptoms of ADs once developed (Gereda et al. 2000, 2001; Park et al. 2001).

#### Biomarkers of susceptibility, exposure, and effect

Several biological markers of nascent hypersensitivity exist (Figure 2). These include IgE, Radioallergoabsorbent test (RAST)-positivity, T-helper type-2 (Th2) cytokine pattern predominance. These markers indicate imbalance in the development of the immune system in newborns and young children and early clinical manifestations of ADs including early development of food allergies and atopic dermatitis manifested as skin rashes. Both genetic and environmental factors have been implicated (Miller 2001). One early marker for atopic immunoreactivity in infancy is the presence of IgE antibody to egg or cow’s milk in serum. The first clinical manifestation of atopic immunoreactivity is, typically, atopic dermatitis, with the highest incidence during the first 3 months of life, followed by food allergy (Wahn and von Mutius 2001). Those individuals who develop the early clinical manifestations, including food allergies and atopic dermatitis, are at higher risk for persistent asthma (Martinez et al. 1995). Thus, measure of these markers may be viewed as early indicators of altered structure or function (Figure 2).

The combination of the measurement of multiple biomarkers of susceptibility and effect coupled with better measures of exposure to multiple pollutants is likely to lead to a more complete understanding of the progression of ADs and the increased incidence and prevalence of asthma in modern, industrialized societies. Disaggregation of the effects is still problematic. Despite the presence of measures of early effect, few data have been collected that show the path of causality from source, (e.g., criteria pollutants), through biomarker (e.g., Th2 cytokine path dominance), to the onset of asthma.

### Neurobehavioral end points

#### Background

Several neurobehavioral end points have been linked to environmental exposures (see Supplemental Material, Figure 1, for a graphical depiction of this link; http://www.ehponline.org/docs/2007/9334/suppl.pdf). Some important known behavioral neurotoxins include mercury and lead. However, making specific chemical/exposure links is difficult because the causes of human disease are varied—resulting from a mixture of environmental, lifestyle, socioeconomic, and genetic factors acting over the life time of the individual.

#### Examples of neurobehaviorally active substances with multiple exposure pathways

##### Mercury

Mercury is a neurotoxic substance that can produce a wide range of health effects depending on the amount and timing of exposure (Clarkson 1997; Ratcliffe et al. 1996; U.S. EPA 1997). Mercury is a naturally occurring element found in the earth’s crust, but human activities contribute significantly (an estimated 70%) to the amount of mercury circulating in the environment (Kyerematen and Vesell 1991). Because of its persistence and the large and distributed number of sources, almost everyone will be exposed to low levels of mercury in air, water, or food (U.S. EPA 1997).

Mercury is somewhat persistent in human tissues (U.S. EPA 1997). This makes it feasible to assess human exposure using blood, hair, and urine. Concentrations of mercury in maternal blood, cord blood, and maternal hair have been used to asses the potential for developmental neurobehavioral end points, but there are significant interindividual variabilities in the relationship among blood-to-hair, blood-to-intake, or hair-to-intake ratios (Bartell et al. 2000). In addition to hair and cord blood, other biomarkers are emerging. For example, mercury selectively alters porphyrin metabolism in kidney proximal tubule cells, leading to an altered urinary porphyrin excretion pattern (Woods 1996), thereby offering a biomarker of effect.

##### Lead

Lead is a naturally occurring heavy metal widely distributed in the Earth’s crust and found in soil, surface water, groundwater, and vegetation and animal tissues. The human body has no known biological need for lead, but once taken in by ingestion, inhalation, or dermal contact, lead behaves like calcium in the body and is stored mostly in the bones, with a small fraction in the blood. The levels of lead in the blood reach equilibrium with bone levels, but this often takes months, even years. Short-term high or low rates of intake can disrupt this equilibrium (Chuang et al. 2001).

Exposures to lead occur in occupational and residential settings. Children and most adults in the United States are more likely to experience chronic low-to-moderate-level lead exposure than acute, high-level exposure.

Among the most significant health problems associated with these levels of lead are neurologic development problems. In adults and children, chronic lower/moderate-level lead exposure has been associated with learning deficiencies, memory problems, behavior problems, attention deficit, problems with coordination, anemia, digestive disorders, renal dysfunction, abnormal reproductive function, and possible infertility (Needleman and Bellinger 1991).

The level of lead in the blood is most often used as the measure of the amount of lead in the body. However, blood lead only relates to the lead exposure within the past few months Currently available tests are subject to numerous limitations such as unreliability and lack of sensitivity for values below a few micrograms per deciliter in blood (Table 1.) This limitation is significant because similar blood lead concentrations have recently been shown to induce irreversible neuropsychologic damage in children (Tong et al. 1998). Like mercury, lead persists in the body so that levels in blood, hair, and bone have served as biomarkers of lead exposure. Lead in teeth and bones can be measured with X rays, but this test is not readily available (Table 1.) One factor limiting the progress of lead epidemiology has been the absence of a biomarker of long-term exposure. Therefore, considerable effort has been devoted to developing methods for the *in vivo* measurement of lead deposited in bone.

#### Effects in children

Human diseases have numerous causative factors that result from a mixture of environmental, lifestyle, socioeconomic, and genetic factors acting over the lifetime of the individual. However, in most cases the environment influences, in varying degrees, the initiation, severity, and/or progression of disease (SB 702 Working Group 2004). Children are particularly vulnerable to environmental disease because their bodies are still developing; they are exposed to more contaminants on a body-weight–adjusted basis; and behaviors such as crawling, putting objects in their mouths, and running that can increase their environmental exposures. Among the most serious diseases confronting children in the United States are neurodevelopmental and behavioral disorders. The manifestation of these diverse effects may arise through exposures to compounds such as mercury and lead in that similar (and multiple) effects may be noted for more general exposure to diverse compounds in the environment.

We consider three effects seen in children that may have etiology rooted, at least in part, in environmental exposure: developmental disorders, attention deficit hyperactivity disorder (ADHD), and delinquency. Examination of these effects in conjunction with multiple sources may offer a fruitful pathway for further understanding.

##### Developmental disorders

Although chemical exposure may be one of the most preventable causes of developmental disorders, the scope of this problem has not been well defined, and there have been only limited attempts at concerted research efforts to use biomarkers to assess cumulative exposures for all substances linked to developmental disorders. So the question to consider is “Are the substances currently known to have effective biomarkers likely to be the ones that will be the dominant contributors to developmental end points?”

There has been recent concern that a signature metabolic impairment or “biomarker” in autistic children strongly suggests that these children would be susceptible to the harmful effects of mercury and other toxic chemical exposures (Parker et al. 2004). To address this issue, the Centers for Disease Control and Prevention conducted its own epidemiologic study and then convened a panel of the Institute of Medicine (IOM) of the National Academy of Sciences to review the issue independently. On 17 May 2004, the IOM published its final report on the possible link between thimerosal and autism and concluded that neither the mercury-based vaccine preservative thimerosal nor the measles–mumps–rubella (MMR) vaccine are associated with autism (IOM 2004). These findings remain controversial, and biomarkers could play a role in further confirming or challenging this finding.

##### Attention deficit hyperactivity disorder

The causes of ADHD are currently not known, but the disorder appears to involve both genetic and environmental components. Possible causes of ADHD include physical trauma to the brain, problems during pregnancy or delivery, genetic abnormalities, and differences in brain structure (National Institute of Mental Health 2005) in addition to or in concert with environmental exposures. There is evidence that environmental exposures can cause problems with behavior and attention similar to those seen in children with ADHD. However, there is little evidence that environmental exposures clearly cause ADHD. For example, many human studies demonstrate that children with elevated lead levels have problems with behavior, impulsivity, and concentration. Several individual case studies suggest that lead exposure may be linked with ADHD. But these studies do not clearly show that lead exposure causes ADHD. Similarly, several human studies suggest that children exposed to methylmercury have an increased risk of behavioral problems. However, this finding is controversial. Not all studies support this association (NRC 2000).

##### Delinquency

Lead exposure has been associated with increased risk for antisocial and delinquent behavior, and the effect follows a developmental course (Needleman et al. 1996). This association links elevated bone lead concentrations and the risk of being arrested for criminal behavior. The underlying causes for this association are not certain. One possibility is that lead interferes with impulse control and that people who have difficulty controlling impulses are more likely to engage in criminal behavior. Another possibility arises from lead’s well-established impact on cognitive function and classroom performance.

In Supplemental Material (http://www.ehponline.org/docs/2007/9334/suppl.pdf), we present an additional case study (“Endocrine Disruptors—An Ecological Point of View”) that uses an ecosystem as a potential sentinel biomarker.

## Summary and Recommendations

In this article, we have identified two focus points for evaluating the capabilities of bio-monitoring data: *a*) Under what circumstances can biomarkers be used to disaggregate disease burden into specific risk factors? and *b*) When and how can biomarkers be used to infer the source and magnitude of exposure among a set of competing sources and pathways? In the following section, we use the case studies above as well as information in the literature on the availability and use of biomarkers to evaluate the capabilities of bio-markers to address these two focus points for cumulative risk assessments.

### Use of biomarkers to disaggregate disease burden into specific risk factors

The use of biomarkers to disaggregate disease burden into specific risk factors is an extension of classic epidemiologic methods, with biomarkers used in place of, or together with, other classification factors. Here, the role of biomarkers is to improve resolution in classifying observed disease occurrence by providing factors that are more explicitly causative or explanatory. This allows for the greatest opportunity of learning to integrate markers of exposure with markers of effect and markers of susceptibility.

Some existing studies have demonstrated the clear advantage of biomarkers for sorting out important risk factors, but much remains to be done. For example, with lead and mercury, the blood levels serve as biomarkers of exposure that have been important as risk factors for disease. These risk factors are sufficiently reliable that biomonitoring for lead and mercury have shaped prevention strategies, helped susceptible subpopulations, and improved the scientific basis for health risk estimates. For neurologic effects, blood lead measurements have been used both as markers of exposure and markers of effect because the harm to the neurologic system depends on the amount of circulating lead in the body. Cotinine has proved useful as a biomarker of exposure to environmental tobacco smoke but is not optimum as a biomarker of effect because cotinine is a metabolite of nicotine that is subject to interindividual variability in measured concentration. To date most efforts to use biomarkers to improve the links among disease patterns and risk factors have been haphazard and opportunistic. In the example of substances as well as the broader range of substances having available biomarkers, there is need for a systematic effort to evaluate whether and how links among exposure and other risk factors can be better tracked to disease burden.

### Use of biomarkers to infer contributions from different sources and pathways

Most environmental pollutants enter human and ecologic receptors from multiple sources and through competing pathways. Effective policies require cumulative risk assessments that not only provide reliable estimates of cumulative intake but also identify the important sources and exposure pathways contributing to this intake. It is common in most risk assessments that this calculation is made in the forward direction—from source to dose. But the increasing availability of population-scale bio-monitoring data makes in possible to work in the opposite direction from dose to source. There have been some preliminary efforts to use biomarkers to infer sources and pathways, but such efforts have been limited. There remains the need for efforts to articulate and evaluate strategies for systematically using biomarkers to infer source and pathway.

The case studies above provide preliminary examples of when and how biomarkers can be used to infer the source and magnitude of exposure among a set of competing sources and pathways. The answer to this question is chemical specific and relates to how well the biomarker matches the characteristics of an “ideal” biomarker, in particular, ease of collection and persistence. For example, the dioxins and polychlorinated biphenyls, which are persistent in biological organisms, facilitate bio-monitoring that has enabled scientists and public health professionals to track population trends and to evaluate progress in reducing exposures. To some extent, biomarkers of dioxin-like substances have also been useful in demonstrating for these compounds the relative importance of global versus regional and local source as well as important contributions through food rather than inhalation pathways. In contrast, biomarkers of a compound that is metabolized relatively quickly provide only limited opportunity for inferring sources or exposure pathways. Biomarkers for OPs are somewhere between the extremes of dioxin-like compounds and rapidly metabolized compounds in providing an opportunity to explore source and pathway contributions. OPs can be measured directly in blood (and possibly in urine) and produce both generic metabolites—DAPs—and OP-specific (or near-specific) biomarkers such as TCPy. The use of these biomarkers in combination provides a better opportunity to disaggregate both source and pathway contributions than is possible for a rapidly metabolized compound. However, little has been done to explore the capabilities and limitations of using multiple biomarkers in combination to infer exposure attributes. One example discussed is the direct intake of the biomarker itself giving rise to an over-estimate of exposure; there are likely others. Important goals for near-term biomarker research must include systematic efforts across a broad range of chemical substances to determine the reliability of biomarkers to infer the source and exposure pathway in cumulative risk assessments. This may be done most effectively through the simultaneous collection of biomarker data of various types on the same individuals, populations, or ecosystems.

In conclusion, the public health goal of quantifying the burden of disease that is attributable to the cumulative impacts of environmental exposure remains elusive. However, the steady progress in development of bio-markers of exposure, susceptibility, and effect, coupled with emerging technologies for environmental monitoring, offers unprecedented opportunities to examine and prevent cumulative health risks and to redefine approaches to environmental protection.

## Figures and Tables

**Table 1:** A comparison of some biomarkers to an ideal biomarker.

Biomarker	Ideal biomarker K	Cotinine	Blood lead	Serum lead	Bone lead
Associated exposure					
Type	Compound K	Nicotine	Lead	Lead	Lead
Exposure	Yes	Yes	Yes	Yes	Yes
Effect	Yes	No	Indicative, not definitive	Yes	Not known
Susceptibility	Indicates specific response to compound K	No	No	No	No
Evaluation characteristics					
Persistence	Yes	No	No	No	Yes
Ease of collection	Readily collected	Readily collected	Readily collected	Somewhat difficult	Difficult
Link to disease	Direct between exposure, source, and disease	Unclear. Cotinine is a marker for smoking, but has not been implicated as a causative agent	Established link between exposure to lead and neurologic disease	Established link between exposure to lead and neurologic disease	No direct link. Endogenous source of lead from bone may be important
Large sample	Large percentage of population carries biomarker	Those who smoke or are exposed to ETS	Yes	Yes	Yes
Broad spatial distribution	Occurs across racial and geographic boundaries	Those who smoke or are exposed to ETS	Yes	Yes	Yes
Appropriate temporal	Occurs over time scales associated with progression of disease	No, short half-life for carcinogenesis end point	No, recent past exposure	No, recent past exposure	Yes
Sensitivity	Displays dose response	Unclear	Some indications	Some indications	Not known, few studies
Parent compound	Directly measures K	Closely associates with nicotine	Direct measure of lead	Direct measure of lead	Direct measure of lead

Abbreviations: ETS, environmental tobacco smoke; K, hypothetical compound for which an ideal biomarker is available.

**Table 2:** Asthma: example evaluation for the outcome.

	Biomarkers and health metrics associated with asthma			
Characteristics of an ideal biomarker	IgE RAST-positivity	Th2 cytokine pattern predominance	Skin rashes	Food allergies
Persistence	Good	Good	Irregular	Good
Ease of collection	Difficult	Difficult	Clinical evaluation	Clinical evaluation
Link to disease	Possibly	Possibly	Possibly	Possibly
Large sample	Yes	Yes	Unknown	Unknown
Broad spatial distribution	Yes	Yes	Unknown	Unknown
Appropriate temporal occurrence	Yes	Yes	Possibly	Possibly
Sensitivity	No	No	Possibly	Possibly
Parent compound	NA	NA	NA	NA

Abbreviations: NA, not applicable; RAST, radioallergoabsorbent test; Th2, T-helper 2.

## References

[b1-ehp0115-000833] Andrae S, Axelson O, Bjorksten B, Fredriksson M, Kjellman NI (1988). Symptoms of bronchial hyperreactivity and asthma in relation to environmental factors. Arch Dis Child.

[b2-ehp0115-000833] Arshad SH, Hide DW (1992). Effect of environmental factors on the development of allergic disorders in infancy. J Allergy Clin Immunol.

[b3-ehp0115-000833] Barr D, Wang R, Needham LL (2005a). Biologic Monitoring of exposure to environmental chemicals throughout the life stages: requirements and issues for consideration for the National Children’s Study. Environ Health Perspect.

[b4-ehp0115-000833] Barr DB, Allen R, Olsson AO, Bravo R, Caltabiano LM, Montesano A (2005b). Concentrations of selective metabolites of organophosphorus pesticides in the United States population. Environ Res.

[b5-ehp0115-000833] Barr DB, Bravo R, Weerasekera G, Caltabiano LM, Whitehead RD, Olsson AO (2004). Concentrations of dialkyl phosphate metabolites of organophosphorus pesticides in the US population. Environ Health Perspect.

[b6-ehp0115-000833] Bartell S, Ponce R, Sanga N, Faustman E (2000). Human variability in mercury toxicokinetics and steady state biomarker ratios. Environ Res.

[b7-ehp0115-000833] Behrendt H, Becker WM, Fritzsche C, Sliwa-Tomczok W, Tomczok J, Friedrichs KH (1997). Air pollution and allergy: experimental studies on modulation of allergen release from pollen by air pollutants. Int Arch Allergy Immunol.

[b8-ehp0115-000833] Callahan MA, Sexton K (2007). If cumulative risk assessment is the answer, what is the question?. Environ Health Perspect.

[b9-ehp0115-000833] Chuang H, Schwartz J, Gonzales-Cossio T, Cortez-Lugo M, Palazuelos E, Aro A (2001). Interrelations of lead levels in bone, venous blood, and umbilical cord blood with exogenous lead exposure through maternal plasma lead in peripartum women. Environ Health Perspect.

[b10-ehp0115-000833] Clarkson T (1997). The toxicology of mercury. Crit Rev Clin Lab Sci.

[b11-ehp0115-000833] Costa LG (2006). Current issues in organophosphate toxicology. Clin Chim Acta.

[b12-ehp0115-000833] Costa LG, Cole TB, Vitalone A, Furlong CE (2005). Measurement of paraoxonase (PON1) status as a potential biomarker of susceptibility to organophosphate toxicity. Clin Chim Acta.

[b13-ehp0115-000833] deFur PL, Evans GW, Cohen Hubal EA, Kyle AD, Morello-Frosch RA, Williams DR (2007). Vulnerability as a function of individual and group resources in cumulative risk assessment. Environ Health Perspect.

[b14-ehp0115-000833] Dejmek J, Selevan SG, Benes I, Solansky I, Sram RJ (1999). Fetal growth and maternal exposure to particulate matter during pregnancy. Environ Health Perspect.

[b15-ehp0115-000833] Diaz-Sanchez D, Tsien A, Fleming J, Saxon A (1997). Combined diesel exhaust particulate and ragweed allergen challenge markedly enhances human in vivo nasal ragweed-specific IgE and skews cytokine production to a T helper cell 2-type pattern. J Immunol.

[b16-ehp0115-000833] Emberlin J (1995). Interaction between air pollutants and aero-allergens. Clin Exp Allergy.

[b17-ehp0115-000833] Gereda JE, Klinnert MD, Price MR, Leung DY, Liu AH (2001). Metropolitan home living conditions associated with indoor endotoxin levels. J Allergy Clin Immun.

[b18-ehp0115-000833] Gereda JE, Leung DY, Thatayatikom A, Streib JE, Price MR, Klinnert MD (2000). Relation between house-dust endotoxin exposure, type 1 T-cell development, and allergen sensitisation in infants at high risk of asthma [See Comment]. Lancet.

[b19-ehp0115-000833] Glenn BS, Stewart WF, Links JM, Todd AC, Schwartz BS (2003). The longitudinal association of lead with blood pressure. Epidemiology.

[b20-ehp0115-000833] Groopman JD, Kensler TW (2005). Role of metabolism and viruses in aflatoxin-induced liver cancer. Toxicol Appl Pharmacol.

[b21-ehp0115-000833] Harving H, Dahl R, Molhave L (1991). Lung function and bronchial reactivity in asthmatics during exposure to volatile organic compounds. Am Rev Respir Dis.

[b22-ehp0115-000833] IOM 2004. Immunization Safety Review: Vaccines and Autism. Washington DC:Institute of Medicine, National Research Council, Immunization Safety Review Committee, National Academies Press. 20669467

[b23-ehp0115-000833] Knox RB, Suphioglu C, Taylor P, Desai R, Watson HC, Peng JL (1997). Major grass pollen allergen Lol p 1 binds to diesel exhaust particles: implications for asthma and air pollution. Clin Exp Allergy.

[b24-ehp0115-000833] Kulig M, Bergmann R, Klettke U, Wahn V, Track U, Wahn U (1999). Natural course of sensitization to food and inhalant allergens during the first 6 years of life. J Allergy Clin Immunol.

[b25-ehp0115-000833] Kyerematen GA, Vesell ES (1991). Metabolism of nicotine. Drug Metab Rev.

[b26-ehp0115-000833] Lu JH, Wu LS, Newman J, Faber B, Gan JY (2006). Degradation of pesticides in nursery recycling pond waters. J Agric Food Chem.

[b27-ehp0115-000833] Lunn JE, Knowelden J, Roe JW (1970). Patterns of respiratory illness in Sheffield junior schoolchildren. A follow-up study. Br J Prev Soc Med.

[b28-ehp0115-000833] MacIntosh D, Needham L, Hammerstrom K, Ryan PB (1999). A longitudinal investigation of selected pesticide metabolites in urine. J Expo Anal Environ Epidemiol.

[b29-ehp0115-000833] Martinez FD, Wright AL, Taussig LM, Holberg CJ, Halonen M, Morgan WJ (1995). Asthma and wheezing in the first six years of life. The Group Health Medical Associates [See Comment]. N Engl J Med.

[b30-ehp0115-000833] Menzie CA, MacDonnell MM, Mumtaz M (2007). A phased approach for assessing combined effects from multiple stressors. Environ Health Perspect.

[b31-ehp0115-000833] Metcalf SW, Orloff KG (2004). Biomarkers of exposure in community settings. J Toxicol Environ Health Part A.

[b32-ehp0115-000833] Miller AL (2001). The etiologies, pathophysiology, and alternative/complementary treatment of asthma. Alt Med Rev.

[b33-ehp0115-000833] Morgan MK, Sheldon LS, Croghan CW, Jones PA, Robertson GL, Chuang JC (2005). Exposures of preschool children to chlorpyrifos and its degradation product 3,5,6-trichloro-2-pyridinol in their everyday environments. J Expo Anal Environ Epidemiol.

[b34-ehp0115-000833] Murray AB, Morrison BJ (1990). It is children with atopic dermatitis who develop asthma more frequently if the mother smokes. J Allergy Clin Immunol.

[b35-ehp0115-000833] National Institute of Mental Health 2005. What Causes ADHD? Bethesda MD:National Institute of Mental Health. Available: http://www.healthyplace.com/Communities/ADD/nimh/causes.htm [accessed 16 October 2005 ].

[b36-ehp0115-000833] NRC (National Research Council) (1987). Biological markers in environmental health research. Environ Health Perspect.

[b37-ehp0115-000833] NRC (National Research Council), Commission on Life Sciences 2000. Toxicological Effects of Methylmercury. Washington, DC:National Academy Press.

[b38-ehp0115-000833] Neas LM, Dockery DW, Ware JH, Spengler JD, Ferris BG, Speizer FE (1994). Concentration of indoor particulate matter as a determinant of respiratory health in children [See Comment]. Am J of Epidemiol.

[b39-ehp0115-000833] Needleman H, Bellinger D (1991). The health effects of low level exposure to lead. Annu Revi Public Health.

[b40-ehp0115-000833] Needleman H, Riess J, Tobin M, Biesecker G, Greenhouse J (1996). Bone lead levels and delinquent behavior. JAMA.

[b41-ehp0115-000833] Norris G, YoungPong SN, Koenig JQ, Larson TV, Sheppard L, Stout JW (1999). An association between fine particles and asthma emergency department visits for children in Seattle. Environ Health Perspect.

[b42-ehp0115-000833] Park JH, Spiegelman DL, Gold DR, Burge HA, Milton DK (2001). Predictors of airborne endotoxin in the home. Environ Health Perspect.

[b43-ehp0115-000833] Parker S, Schwartz B, Todd J, Pickering L (2004). Thimerosal-containing vaccines and autistic spectrum disorder: a critical review of published original data. Pediatrics.

[b44-ehp0115-000833] Peterson B, Saxon A (1996). Global increases in allergic respiratory disease: the possible role of diesel exhaust particles. Ann Allergy Asthma Immunol.

[b45-ehp0115-000833] Platts-Mills TA, Woodfolk JA, Chapman MD, Heymann PW (1996). Changing concepts of allergic disease: the attempt to keep up with real changes in lifestyles. J Allergy Clin Immunol.

[b46-ehp0115-000833] Plog B Niland J Quinlan P eds. 1996. Fundamentals of Industrial Hygiene. Itasca, IL:National Safety Council.

[b47-ehp0115-000833] Ratcliffe H, Swanson G, Fischer L (1996). Human exposure to mercury: a critical assessment of the evidence of adverse health effects. J Toxicol Environ Health.

[b48-ehp0115-000833] Romieu I, Meneses F, Ruiz S, Sienra JJ, Huerta J, White MC (1996). Effects of air pollution on the respiratory health of asthmatic children living in Mexico City. Am J Respir Crit Care Med.

[b49-ehp0115-000833] Rubbin J, Liu M, Sessoms R, Banerjee S, Banerjee U (1986). Effects of certain atmospheric pollutants on the soluble amino acides, molecular weight and antigenicity of some airborne pollen grains. Cytobios.

[b50-ehp0115-000833] Ruffin J, Liu MY, Sessoms R, Banerjee S, Banerjee UC (1986). Effects of certain atmospheric pollutants (SO_2_, NO_2_ and CO) on the soluble amino acids, molecular weight and antigenicity of some airborne pollen grains. Cytobios.

[b51-ehp0115-000833] SB 702 Working Group 2004. Strategies for Establishing an Environmental Health Surveillance System in California A Report of the SB 702 Expert Working Group. Berkeley, CA:The California Policy Research Center.

[b52-ehp0115-000833] Schulte P, Mazzuckelli LF (1991). Validation of biological markers for quantitative risk assessment. Environ Health Perspect.

[b53-ehp0115-000833] Sexton K, Hattis D Assessing cumulative health risks from exposure to environmental mixtures—three fundamental questions. Environ Health Perspect.

[b54-ehp0115-000833] Stewart WF, Schwartz BS, Davatzikos C, Shen D, Liu D, Wu X (2006). Past adult lead exposure is linked to neurodegeneration measured by brain MRI. Neurology.

[b55-ehp0115-000833] Tong S, Baghurst P, Sawyer M, Burns J, McMichael A (1998). Declining blood lead levels and changes in cognitive function during childhood: The Port Pirie Cohort Study. JAMA.

[b56-ehp0115-000833] U.S. EPA 1997. U.S. Environmental Protection Agency Mercury Study Report to Congress, EPA-452/R-97-003. Washington, DC:U.S. Environmental Protection Agency.

[b57-ehp0115-000833] U.S. EPA 2003. Framework for Cumulative Risk Assessment. Washington, DC:U.S. Environmental Protection Agency.

[b58-ehp0115-000833] Ussetti P, Roca J, Agusti AG, Montserrat JM, Rodriguez-Roisin R, Agusti-Vidal A (1984). Another asthma outbreak in Barcelona: role of oxides of nitrogen. Lancet.

[b59-ehp0115-000833] Wahn U, von Mutius E (2001). Childhood risk factors for atopy and the importance of early intervention [See Comment]. J Allergy Clin Immunol.

[b60-ehp0115-000833] Ware JH, Ferris BG, Dockery DW, Spengler JD, Stram DO, Speizer FE (1986). Effects of ambient sulfur oxides and suspended particles on respiratory health of preadolescent children. Am Rev Respir Dis.

[b61-ehp0115-000833] WHO 1993. Biomarkers and Risk Assessment: Concepts and Principles. Geneva:World Health Organization.

[b62-ehp0115-000833] Wilson NK, Chuang JC, Iachan R, Lyu C, Gordon SM, Morgan MK (2004). Design and sampling methodology for a large study of preschool children’s aggregate exposures to persistent organic pollutants in their everyday environments. J Expo Anal Environ Epidemiol.

[b63-ehp0115-000833] Woods J (1996). Altered porphyrin metabolism as a biomarker of mercury exposure and toxicity. Can J Physiol Pharmacol.

